# Prevalence of Primary Dysmenorrhoea and Its Impact on Academic Performance among Croatian Students during the COVID-19 Pandemic

**DOI:** 10.1155/2023/2953762

**Published:** 2023-06-03

**Authors:** Marta Horvat, Doroteja Pavan Jukić, Lovro Marinović, Dina Bursać, Rosana Ribić, Marijana Neuberg, Danijel Bursać

**Affiliations:** ^1^Health Center Zagreb West, Zagreb, Croatia; ^2^Department of Obstetrics and Gynecology, University Hospital Merkur, Zagreb, Croatia; ^3^Department of Pathology, University Hospital Merkur, Zagreb, Croatia; ^4^Department of Oral Surgery, University Hospital Centre Zagreb, Zagreb, Croatia; ^5^University North, Varaždin, Croatia

## Abstract

**Background:**

Dysmenorrhoea is one of the most common gynaecological problems. Therefore, it is important to investigate its impact during the COVID-19 pandemic which has a great impact on the lives of menstruating people all over the world.

**Aim:**

To determine the prevalence and impact of primary dysmenorrhoea on academic performance among students during the pandemic.

**Materials and Methods:**

This cross-sectional study was conducted in April 2021. All data were collected by an anonymous self-assessed web‐based questionnaire. Due to voluntary participation in the study, 1210 responses were received, but 956 were left for analysis after exclusion criteria were applied. Descriptive quantitative analysis was performed and Kendall rank correlation coefficient was used.

**Results:**

The prevalence of primary dysmenorrhoea was 90.1%. Menstrual pain was mild in 7.4% of cases, moderate in 28.8%, and severe in 63.8%. The study found that primary dysmenorrhoea has a great perceived impact on all included aspects of academic performance. Most affected were concentration in class in 810 (94.1%) and doing homework and learning in 809 (94.0%) female students. There is also a correlation between menstrual pain intensity and its impact on academic performance (*p* < 0.001).

**Conclusions:**

Our study found that the prevalence of primary dysmenorrhoea among students at the University of Zagreb is high. Painful menstruation greatly impacts academic performance and therefore it is important to do more research on this topic.

## 1. Introduction

Painful menstruation or dysmenorrhoea is chronic, cyclic pelvic pain associated with menstruation [1]. Primary dysmenorrhoea denotes cramping in the lower part of the abdomen which occurs just before or within a few hours after the beginning of menstruation and in absence of pelvic pathology [2]. Its onset is 6–12 months after menarche which is associated with the establishment of ovulatory cycles, and it reaches its peak in late adolescence [3]. Other symptoms that may accompany primary dysmenorrhoea include back and thigh pain, headache, nausea, diarrhoea, and vomiting. Secondary dysmenorrhoea can occur at any time during a woman's reproductive period, but it is most commonly more than two years after menarche. Depending on the underlying condition, it may be accompanied by other gynaecological symptoms such as intermenstrual bleeding, menorrhagia, or dyspareunia. The most common cause of secondary dysmenorrhoea is endometriosis [4].

According to previous studies, the prevalence of primary dysmenorrhoea in women of reproductive age is between 45% and 95% [5]. Severe pain or pain that significantly limits their daily activities is experienced by 2–28% of women [6]. Primary dysmenorrhoea is associated with reduced quality of life; it has an impact on family relationships, friendships, social, and sports activities and often causes absenteeism from school, university, or work and a decline in academic performance [7, 8]. A negative impact of primary dysmenorrhoea is observed on many aspects of academic performance which include class attendance, homework writing, exam passing, and grades, but according to some studies, concentration in class is most affected [9, 10]. Previous longitudinal studies show that absenteeism rates due to primary dysmenorrhoea in young women are between 34% and 50% and estimate that 10–30% of all female students and employed young women with dysmenorrhoea lose 1-2 working days per month [11, 12]. The need to take a day off results in a loss of productivity which ultimately leads to economic losses as evidenced by data from some older studies, e.g., an annual loss of 2.6 billion dollars was estimated in Japan [13, 14].

Although painful menstruation is a significant gynaecological problem in young women all over the world, there is no data about its prevalence and effect on the academic performance of Croatian university students. Therefore, the objective of the present study was to estimate the prevalence and impact of primary dysmenorrhoea on the academic performance of female students at the University of Zagreb.

## 2. Materials and Methods

This cross-sectional study was conducted in April 2021. The study protocol was approved by the Ethics Committee at the University of Zagreb School of Medicine (380-59-10106-21-111/66). All of the 34 constituent units of the University of Zagreb were asked for permission to conduct the research among their students, but only 22 (64.7%) answered and gave approval for conducting the survey. Permission to conduct the survey was not obtained from 12 (35.3%) units: 11 (32.4%) did not answer the request and 1 (2.9%) did not want to give permission without the approval of their own Ethics Committee. The data were collected by an anonymous web-based questionnaire. All data were self-reported. Participation in this study was entirely voluntary, and the participants were informed through written consent that their anonymity was assured and they could withdraw at any time.

**Figure 1:**
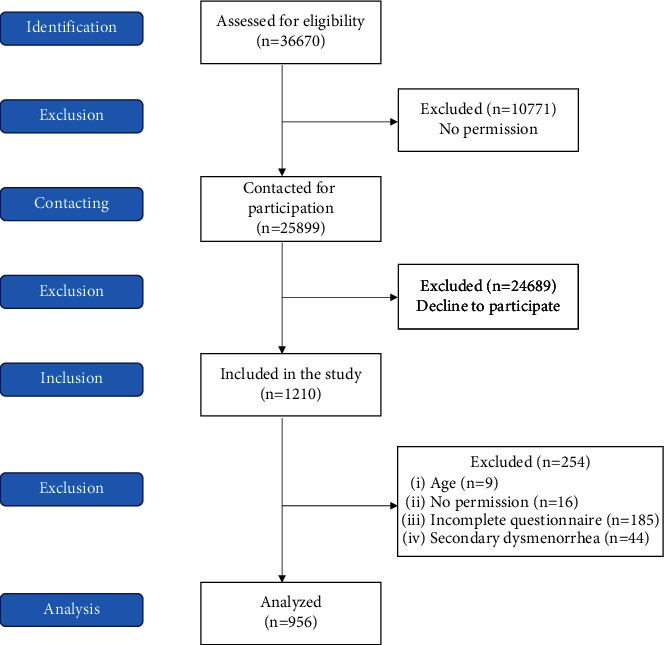
STROBE diagram of study participants.

Inclusion criteria included female sex and enrolment at one of the constituent units of the University of Zagreb during the research period. Exclusion criteria included females aged above 30 years and pelvic pathology connected with secondary dysmenorrhoea (e.g., endometriosis, adenomyosis, pelvic inflammatory disease, cervical stenosis, cervical polyps, and uterine fibroids). As some constituent units did not give their permission to conduct the research, responses from students who were enrolled there could not be included as well. The STROBE diagram of study participants is presented in Figure 1.

Data were collected through a self-report questionnaire designed based on the available literature and questionnaires used in previous studies [9, 15]. The questionnaire was developed on the Google Forms platform, and it contained sociodemographic questions and questions regarding the menstrual cycle and the perceived influence of menstrual pain on academic performance. A Likert scale (0–3) was used to estimate the perceived influence of dysmenorrhoea on academic performance. Dysmenorrhoea was defined as painful menstruation at least once in the preceding six months [16, 17]. The menstrual bleeding pattern was estimated by the number of pads or tampons used during one menstruation. Thus, light bleeding corresponded to the use of ≤10 pads/tampons, moderate 11–20 pads/tampons, moderately heavy 21–30 pads/tampons, and heavy more than 30 pads/tampons during one menstrual cycle [18]. The intensity of pain from 0 to 10 during menstruation was examined using a numerical rating scale (NRS), and the score was interpreted as in previous studies: mild [1–3], moderate [4–6], and severe [7–10] [19].

A pilot study was conducted among 20 female students, whose answers were later not included in the analysis, to determine the comprehensibility of individual questions. The questions that proved unclear were subsequently supplemented by additional instructions and explanations.

An invitation mail including the link to our study questionnaire was sent to student representatives of all university units which gave approval for conducting the survey. They mostly distributed the questionnaire on official pages of student councils or groups on social media (e.g., Facebook and Instagram) and a smaller part through students' official e-mail addresses. Every student with a Facebook and/or Instagram account could access our questionnaire. As the participation in this study was anonymous, we did not gather any personal information and could not identify repeated attempts of survey response.

Categorical variables were summarized as counts and percentages, and ordered or continuous variables as mean, standard deviation, minimum, and maximum. Correlation between ordinal variables was determined using the Kendall rank correlation coefficient. Statistical analysis was performed using scripts written in Python 3.9.

## 3. Results

**Figure 2:**
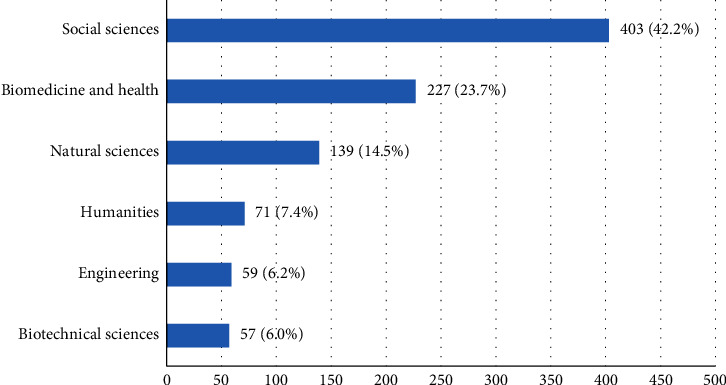
Student's distribution according to their field of study at the University of Zagreb.

A total of 1210 Croatian female students completed an anonymous web-based questionnaire. After exclusion criteria were applied, 254 responses were dismissed: 9 (3.5%) students aged above 30 years, 16 (6.3%) students whose institutions did not approve the study and accidentally gained access to the questionnaire, 185 (72.8%) students submitted an incomplete questionnaire, and 44 (17.3%) students were diagnosed with one of the possible causes of secondary dysmenorrhoea. A total of 956 responses remained for analysis.  Figure 2 shows the student distribution according to their field of study.

Sociodemographic and menstrual characteristics of the participating students are presented in Table 1.

The study found a prevalence of primary dysmenorrhoea of 90.1% (95% CI 88.0%, 91.9%). In total, 861 students reported the experience of painful menstruation at least once in the last six months. The mean intensity of menstrual pain was 6.77 (1.86) (range 0–10 per NRS). Mild pain was reported by only 64 female students, or 7.4%, while 248 (28.8%) had moderate pain and 549 (63.8%) reported severe pain.

Table 2 shows the relation between the intensity of primary dysmenorrhoea and its perceived impact on academic activities which include class attendance, concentration in class, doing homework, and learning as well as exam performance.

The study found that painful menstruation affects concentration in class in 806 (93.6%) students and doing homework and learning in 803 (93.3%). Exam performance is limited due to dysmenorrhoea in 750 (87.1%) students. Dysmenorrhoea has also an impact on class attendance or absenteeism in 590 (68.5%) students.

## 4. Discussion

The prevalence of primary dysmenorrhoea was 90.1%, which is slightly more than other studies conducted among women in the same age group. The prevalence of menstrual pain according to these studies is 88.0% in Australia [16], 76.7% in Ethiopia [20], 84.1% in Italy [21], 89.1% in Malesia [22], 64.9% in Poland [23], 80.0% in Saudi Arabia [24], 84.8% in Serbia [25], 76.5% in Spain [8], and 55.5–88.0% in Turkey [7, 26–28]. Differences arise due to the use of different methods of data collection such as self-reported physical or online questionnaires, but also through a conversation with research participants (interview) conducted live or by phone. In addition, sample size and criteria for inclusion of participants play a significant role. The most important factor contributing to the differences in established prevalence is the lack of a unified definition of primary dysmenorrhoea. In some studies [16], dysmenorrhoea was defined as painful menstruation regardless of intensity, while others [21] consider dysmenorrhoea only menstrual pain associated with the need to take medication and a significant limitation of normal activities, or pain so strong that women have to stay at home or in bed. The high prevalence of dysmenorrhoea in our study could be the result of a selection bias because female students who have painful menstruation could be more motivated to respond to such a questionnaire.

The average intensity of menstrual pain in this study was 6.77 (1.86), which is consistent with the results of other conducted studies [29, 30]. Mild pain was recorded in 64 female students (7.4%), 248 (28.8%) had moderate pain and 549 (63.8%) had severe pain. The number of students who reported having severe pain was significantly higher than that of other studies [21–24, 31], in which between 27.0% and 40.1% of young women had very painful menstruation. When comparing pain intensity recorded in our study to previous studies, it should be taken into account that in most of the studies mentioned, different pain scales were used to assess the intensity of dysmenorrhoea, and pain scores were also interpreted differently in some other studies [7, 19].

This study was the first to report the prevalence and impact of primary dysmenorrhoea among female students at the University of Zagreb, Croatia. It is important to notice that it was conducted during the peak of the second wave of the COVID-19 pandemic in Croatia. Furthermore, while in coronavirus lockdown, Zagreb was hit by an earthquake [32] and another destructive earthquake with its epicentre located roughly 50 km from Zagreb [33] happened less than six months before the study questionnaire was distributed.

It is observed that the students perceive menstrual pain to have a great impact on their class attendance, concentration in class, doing homework and learning, and exam performance. When comparing these results with those of other studies [15, 34, 35], a significantly greater impact of menstrual pain on academic performance is observed in Croatian students. When considering these results, it should be taken into account that the experience of pain is subjective and dependent on various genetic and sociocultural factors. The results of our study also indicate that the impact of dysmenorrhoea on all observed activities, especially concentration in class and doing homework and learning, is significantly higher in female students with severe pain than those with mild or moderate pain, which is in accordance with the results of previous research [36]. Stressful events such as the COVID-19 pandemic and earthquakes could influence the prevalence and intensity of dysmenorrhoea, as well as the greater perceived impact of menstrual pain on various academic activities of students at the University of Zagreb. Other studies already described a connection between COVID-19 pandemic-related psychological distress and menstrual symptoms [37, 38].

Data gathered in this study could be used to arrange campaigns and increase people's awareness about dysmenorrhoea and its effect on academic and work performance in women. Moreover, it would be useful to organize public health campaigns, for example, small workshops for students to share their experience and learn together how to decrease menstrual pain in a way which is most effective for every individual in accordance with her needs. Furthermore, every student should have the chance to seek advice from a gynaecologist, although their chosen gynaecologist is not in the town they are studying in.

Although our study tried to reach most of the students enrolled at the University of Zagreb, we could not include students enrolled at units which did not gave approval to conduct the survey. Some of these units are rather conservative and for this reason, the answers of their students could differ from our sample. As participation was voluntary, students who are not affected with dysmenorrhoea could also have no interest in completing our questionnaire, which could cause a bias which is discussed in the next paragraph.

Most participants (42.2%) were enrolled in social sciences and least in engineering (6.2%) and biotechnical sciences (6.0%). The distribution of participants relating to their field of study is mostly in accordance with the distribution of students at the University of Zagreb [39] which makes the sample more representative. Limitations in this research are primarily due to its cross-sectional structure, as well as voluntary participation and completion of a self-reported questionnaire by female students. The definition of dysmenorrhoea used in this study, painful menstruation at least once in the last six months, may not have been adequate and a more precise definition should be used in future studies. As mentioned earlier, female students completed the questionnaire voluntarily, and the high prevalence of dysmenorrhoea could be due to a volunteer bias because female students with painful menstruation were more likely to complete the study questionnaire. Although an exclusion criterion was used to identify female students with secondary dysmenorrhoea, it could be that some of the students who reported to have primary dysmenorrhoea have a pelvic pathology associated with secondary dysmenorrhoea which is not diagnosed or they have never been on a gynaecological exam and their physical condition is unknown. There are other important confounding factors which could have an influence on the occurrence of dysmenorrhoea (e.g., body mass index, smoking, socioeconomic status, use of oral contraceptives, and parity) which have not been taken into consideration.

## 5. Conclusion

This study found a high prevalence of primary dysmenorrhoea among students enrolled at the University of Zagreb. Furthermore, it showed that menstrual pain has a significant impact on their academic performance. Students who suffer more painful periods are at greater risk to have decreased academic performance. More research is needed to determine the impact of primary dysmenorrhoea on other aspects of students' lives and pain management, which will eventually help to educate young women about their condition and increase their quality of life.

## Figures and Tables

**Table 1:** Demographic and gynaecological health characteristics of participating students.

Variable	Students *N* = 956
Age (years)	21.8 (2.0)
Menarche age (years)	12.5 (1.3)

*Regular menstrual cycle*	
Yes	773 (80.9)
No	183 (19.1)
Menstrual cycle duration (days)	5.6 (1.0)

*Heaviness of menstrual flow*	
Light	60 (6.3)
Moderate	508 (53.1)
Moderately heavy	327 (34.2)
Heavy	61 (6.4)

*Family history of dysmenorrhoea*	
Yes	713 (74.6)
No	243 (25.4)

**Table 2:** Relationship between the intensity of dysmenorrhoea and its perceived impact on some academic activities.

Activity Impact	Intensity of dysmenorrhoea			Kendall tau	95% CI
	Mild *N* = 64 *n* (%)	Moderate *N* = 248 *n* (%)	Severe *N* = 549 *n* (%)		
*Class attendance*					
None	51 (79.7)	111 (12.9)	109 (12.7)	0.444	0.408–0.479
Mild	10 (15.6)	78 (9.1)	119 (13.8)		
Moderate	3 (4.7)	53 (6.1)	201 (23.3)		
Major	0 (0.0)	6 (0.7)	120 (13.9)		

*Concentration in class*					
None	27 (42.2)	23 (2.7)	5 (0.6)	0.494	0.460–0.527
Mild	28 (43.8)	93 (10.8)	70 (8.1)		
Moderate	8 (12.5)	104 (12.1)	211 (24.5)		
Major	1 (1.6)	28 (3.3)	263 (30.5)		

*Doing homework/learning*					
None	26 (40.6)	23 (2.7)	8 (0.9)	0.446	0.410–0.481
Mild	29 (45.3)	92 (10.7)	65 (7.5)		
Moderate	8 (12.5)	94 (10.9)	227 (26.4)		
Major	1 (1.6)	39 (4.5)	249 (28.9)		

*Exam performance*					
None	35 (54.7)	46 (5.3)	30 (3.5)	0.437	0.400–0.472
Mild	24 (37.5)	110 (12.8)	109 (12.7)		
Moderate	4 (6.3)	64 (7.4)	225 (26.1)		
Major	1 (1.6)	28 (3.3)	185 (21.5)		

^†^Percentages may not total 100% due to rounding.

## Data Availability

The data used to support the findings of this study are currently under embargo while the research findings are commercialized. Requests for data, 12 months after publication of this article, will be considered by the corresponding author.

## Supplementary Materials

Supplementary Materials Questionnaire: informed consent and questionnaire used in the study.
