# Supplementary material for: Prevalence of Primary Dysmenorrhoea and Its Impact on Academic Performance among Croatian Students during the COVID-19 Pandemic
Source: Obstet Gynecol Int. 2023 Jun 3;2023:2953762. doi: 10.1155/2023/2953762 (PMC10257551; doi:10.1155/2023/2953762)
Supplement: Supplementary Materials — Questionnaire: informed consent and questionnaire used in the study. [file 2953762.f1.pdf]

Prevalencija i utjecaj primarne dismenoreje na kvalitetu života studentica Sveučilišta u Zagrebu

Poštovane studentice,

pozivamo Vas na sudjelovanje u istraživanju pod nazivom „Prevalencija i utjecaj primarne dismenoreje na kvalitetu života studentica Sveučilišta u Zagrebu“. Cilj ovog istraživanja je utvrditi učestalost primarne dismenoreje u populaciji studentica Sveučilišta u Zagrebu i ispitati njezin utjecaj na kvalitetu njihova života. Primarna dismenoreja (bolna menstruacija) označava bolne grčeve u donjem dijelu trbuha koji se javljaju neposredno prije i/ili tijekom menstruacije, a u odsustvu poznate zdjelične bolesti. Uobičajeno se počinje javljati tijekom adolescencije, ubrzo nakon prve menstruacije, a jačina bolova može varirati u rasponu od vrlo blagih do jakih i onesposobljavajućih. U ranije provedenim istraživanjima utvrđeno je kako menstrualni bolovi mogu ometati svakodnevne aktivnosti i imaju negativan utjecaj na mnoge aspekte života zahvaćenih djevojaka i žena koji uključuju obiteljske odnose, školsku/akademsku izvedbu, društvene aktivnosti i rekreaciju.

Istraživanje je odobrilo Etičko povjerenstvo Medicinskog fakulteta Sveučilišta u Zagrebu i provodi se uz poštivanje svih etičkih načela. Sudjelovanje u ovom istraživanju je potpuno dobrovoljno i možete odustati od njega u bilo kojem trenutku. Zamolit ćemo Vas da ispunite online upitnik u kojem su pitanja podijeljena u nekoliko skupina. Pitanja na koja je odgovor obavezan označena su crvenom zvjezdicom, a ispod pitanja u kojima se koristi stručna terminologija navedeno je kratko objašnjenje. Upitnik je u cijelosti anoniman što znači da se odgovori ni na koji način neće moći povezati s Vama. Nema točnih i netočnih odgovora, stoga Vas molimo da na sva pitanja odgovorite iskreno. Rizika vezanih uz samo sudjelovanje nema, pitanja nisu osjetljive prirode niti je predviđeno da Vas na bilo koji način uznemire. Korist ovog istraživanja je u tome što će se pomoću njega ukazati na potrebu za informiranjem i smanjenjem stigmatizacije menstrualnih poremećaja. Svi prikupljeni podaci obrađivat će se na grupnoj razini i bit će korišteni samo u znanstvene svrhe. Za ispunjavanje upitnika potrebno je izdvojiti 5-10 minuta.

Pritiskom na "Dalje" dajete svoju suglasnost za sudjelovanje u istraživanju i potvrđujete da ste upoznati s uvjetima sudjelovanja.

*Obavezno

Demografski podaci

1. Koliko imate godina? *

____

2. Označite sastavnicu Sveučilišta u Zagrebu na kojoj studirate. *

Označite samo jedan oval.

- Agronomski fakultet
- Akademija dramske umjetnosti
- Akademija likovnih umjetnosti
- Arhitektonski fakultet
- Edukacijsko-rehabilitacijski fakultet
- Ekonomski fakultet
- Fakultet elektrotehnike i računarstva
- Fakultet filozofije i religijskih znanosti
- Fakultet hrvatskih studija
- Fakultet kemijskog inženjerstva i tehnologije
- Fakultet organizacije i informatike
- Fakultet političkih znanosti
- Fakultet prometnih znanosti
- Fakultet strojarstva i brodogradnje
- Farmaceutsko-biokemijski fakultet
- Filozofski fakultet
- Geodetski fakultet
- Geotehnički fakultet
- Građevinski fakultet
- Grafički fakultet
- Katolički bogoslovni fakultet
- Kineziološki fakultet
- Medicinski fakultet
- Metalurški fakultet
- Muzička akademija
- Pravni fakultet
- Prehrambeno-biotehnološki fakultet
- Prirodoslovno-matematički fakultet
- Rudarsko-geološko-naftni fakultet
- Stomatološki fakultet
- Šumarski fakultet

- Tekstilno-tehnološki fakultet
- Učiteljski fakultet
- Veterinarski fakultet

3. Koji je broj stanovnika Vašeg mjesta prebivališta? *

Označite samo jedan oval.

- manje od 2 000
- 2 000 - 5 000
- 5 000 - 10 000
- 10 000 - 35 000
- više od 35 000

Podaci o menstruacijskom ciklusu

4. S koliko ste godina dobili prvu menstruaciju? *

____

5. Jesu li Vaši menstruacijski ciklusi redoviti? *

Označite samo jedan oval.

- Da
- Ne

6. Koliko dana prosječno traje Vaš menstruacijski ciklus? *

Prvi dan menstruacijskog ciklusa je prvi dan menstrualnog krvarenja. Ako duljina Vašeg menstruacijskog ciklusa varira npr. između 27 i 30 dana, upišite srednju vrijednost, odnosno najčešće trajanje ciklusa, primjerice 29.

____

7. Koliko dana u prosjeku traje Vaše menstruacijsko krvarenje? *

Ako se duljina Vašeg menstruacijskog krvarenja razlikuje od ciklusa do ciklusa, npr. 5-6 dana, upišite koliko najčešće traje, primjerice 5.

____

8. Koliko je obilno Vaše menstruacijsko krvarenje prema broju uložaka/tampona koje iskoristite za vrijeme jedne menstruacije? *

oskudno (manje od 10 uložaka/tampona), umjereno (11-20 uložaka/tampona), umjereno obilno (21-30 uložaka/tampona), obilno (više od 30 uložaka/tampona)

Označite samo jedan oval.

- oskudno
- umjereno
- umjereno obilno
- obilno

9. Jeste li barem jednom u posljednjih 6 mjeseci imali bolne menstruacije? *

Označite samo jedan oval.

- Da
- Ne *Idite na odjeljak 5 (Puno Vam hvala na izdvojenom vremenu! :)).*

10. Ukoliko Vam je dijagnosticirana neka od sljedećih ginekoloških bolesti, označite o čemu se radi ili upišite naziv dijagnoze na za to predviđeno mjesto. *

Odaberite sve točne odgovore.

- [ ] nisam nikad bila kod ginekologa
- [ ] nemam dijagnosticiranu nijednu ginekološku bolest s popisa
- [ ] endometrioza
- [ ] adenomioza
- [ ] zdjelična upalna bolest (PID)
- [ ] stenoza vrata maternice
- [ ] cervikalni polipi
- [ ] miomi
- [ ] Ostalo: _______________________________

11. Je li netko u Vašoj obitelji imao ili ima bolne menstruacije? *

Označite samo jedan oval.

- Da
- Ne

12. Ako je odgovor na prethodno pitanje "Da", označite na koga se to odnosi.

Odaberite sve točne odgovore.

- baka
- majka
- teta
- sestra

Utjecaj na kvalitetu života

13. Označite intenzitet bolova za vrijeme menstruacije. *

Označite kakvog su intenziteta Vaši menstrualni bolovi pri čemu 0 - nema bolova, 10 - najgori mogući bolovi.

Označite samo jedan oval.

0	1	2	3	4	5	6	7	8	9	10

14. Svakodnevne aktivnosti. *

Označite kakav utjecaj menstrualni bolovi imaju na Vaše svakodnevne aktivnosti pri čemu 0 - nema utjecaja, 1 - blagi utjecaj, 2 - umjeren utjecaj i 3 - ozbiljan utjecaj.

Označite samo jedan oval.

0	1	2	3

15. Prisutnost na nastavi. *

Označite kakav utjecaj menstrualni bolovi imaju na Vašu prisutnost na nastavi pri čemu 0 - nema utjecaja, 1 - blagi utjecaj, 2 - umjeren utjecaj i 3 - ozbiljan utjecaj.

Označite samo jedan oval.

0	1	2	3
◯	◯	◯	◯

16. Sposobnost koncentracije na nastavi. *

Označite kakav utjecaj menstrualni bolovi imaju na Vašu sposobnost koncentracije na nastavi pri čemu 0 - nema utjecaja, 1 - blagi utjecaj, 2 - umjeren utjecaj i 3 - ozbiljan utjecaj.

Označite samo jedan oval.

0	1	2	3
◯	◯	◯	◯

17. Rješavanje zadaće i učenje. *

Označite kakav utjecaj menstrualni bolovi imaju na Vaše rješavanje zadaće i učenje pri čemu 0 - nema utjecaja, 1 - blagi utjecaj, 2 - umjeren utjecaj i 3 - ozbiljan utjecaj.

Označite samo jedan oval.

0	1	2	3
◯	◯	◯	◯

18. Izvedba na ispitu. *

Označite kakav utjecaj menstrualni bolovi imaju na Vašu izvedbu na ispitu pri čemu 0 - nema utjecaja, 1 - blagi utjecaj, 2 - umjeren utjecaj i 3 - ozbiljan utjecaj.

Označite samo jedan oval.

0	1	2	3
⬭	⬭	⬭	⬭

19. Obiteljski odnosi. *

Označite kakav utjecaj menstrualni bolovi imaju na Vaše obiteljske odnose pri čemu 0 - nema utjecaja, 1 - blagi utjecaj, 2 - umjeren utjecaj i 3 - ozbiljan utjecaj.

Označite samo jedan oval.

0	1	2	3
⬭	⬭	⬭	⬭

20. Druženje s prijateljima. *

Označite kakav utjecaj menstrualni bolovi imaju na Vaše druženje s prijateljima pri čemu 0 - nema utjecaja, 1 - blagi utjecaj, 2 - umjeren utjecaj i 3 - ozbiljan utjecaj.

Označite samo jedan oval.

0	1	2	3
⬭	⬭	⬭	⬭

21. Odlazak na kulturna događanja (npr. koncerti, kino, izložbe). *

Označite kakav utjecaj menstrualni bolovi imaju na Vaše odlaske na kulturna događanja (npr. koncerti, kino, izložbe) pri čemu 0 - nema utjecaja, 1 - blagi utjecaj, 2 - umjeren utjecaj i 3 - ozbiljan utjecaj.

Označite samo jedan oval.

0	1	2	3
⬭	⬭	⬭	⬭

22. Sudjelovanje u sportskim aktivnostima. *

Označite kakav utjecaj menstrualni bolovi imaju na Vaše sudjelovanje u sportskim aktivnostima pri čemu 0 - nema utjecaja, 1 - blagi utjecaj, 2 - umjeren utjecaj i 3 - ozbiljan utjecaj.

Označite samo jedan oval.

0	1	2	3
⬭	⬭	⬭	⬭

Puno Vam hvala na izdvojenom vremenu! :)

Google nije izradio niti podržava ovaj sadržaj.

Google Obrasci
